# Implementation of WHO Recommended Policies and Interventions on Healthy Diet in the Countries of the Eastern Mediterranean Region: From Policy to Action

**DOI:** 10.3390/nu12123700

**Published:** 2020-11-30

**Authors:** Ayoub Al-Jawaldeh, Asmus Hammerich, Radhouene Doggui, Kaia Engesveen, Krista Lang, Karen McColl

**Affiliations:** 1 WHO Regional Office for Eastern Mediterranean Region, Cairo 11371, Egypt; hammericha@who.int; 2 Department of Family Medicine, Université de Sherbrooke, Sherbrooke, QC J1K 2R1, Canada; doggui.radhouene@gmail.com; 3 Centre de Formation Médicale du Nouveau-Brunswick, Moncton, NB E1A 7R1, Canada; 4 WHO Department of Nutrition and Food Safety, 1211 Geneva, Switzerland; engesveenk@who.int (K.E.); zillmerk@who.int (K.L.); 5 Independent Consultant, West Sussex PO21 4NB, UK

**Keywords:** nutrition, healthy diet, unhealthy diet: noncommunicable diseases, policy, legislation, obesity, overweight

## Abstract

Non-communicable diseases (NCDs) are responsible for almost two-thirds of the deaths in the 22 countries and territories of the WHO Eastern Mediterranean Region and unhealthy diets are a major contributor. Prevalence of overweight and obesity has increased among adults, adolescents and older children in recent decades. Among countries with the highest prevalence there are signs that the increase is slowing down or even that prevalence is declining. There has been no increase in the prevalence rate in younger children, although the absolute number of children under five years affected by overweight has increased. This review summarizes prevalence data and examines current implementation of regulatory, fiscal and voluntary measures to promote healthy diet across the Region. The last decade has seen a step up in such action. Ten of the Region’s countries have policies relating to trans-fatty acids and they are increasingly implementing specific regulatory measures. Thirteen countries had fully or partially implemented national salt reduction policies by 2019. Only four countries had adopted policies relating to aspects of marketing food to children by 2019, and concrete action in this area is still lacking. Eight countries have introduced taxes—sometimes at a rate of 50%—on carbonated or sugar-sweetened beverages. In order to meet the agreed global and regional goals relating to nutrition and diet-related NCDs, countries will need to build on this progress and scale up action across the Region while intensifying efforts in areas where concrete action is lacking.

## 1. Introduction

Most countries in the World Health Organization (WHO) Eastern Mediterranean Region have experienced the nutrition transition towards unhealthy diets and sedentary lifestyles, and almost half the Region’s adults (49%), over a quarter (26%) of adolescents and nearly 6% of children under five are affected by overweight or obesity. Noncommunicable diseases (NCDs) are responsible for 62% of all deaths in the Region and unhealthy diet is a major contributor [1]. Most countries in the Region are faced with the double burden of malnutrition, whereby undernutrition and overweight/obesity co-exist within the population [1]. Across the Region, unhealthy dietary practices are prevalent in children, adolescents and adults [2,3].

Healthy diets help to protect against malnutrition in all its forms, as well as diet-related NCDs. Healthy dietary practices start early in life, and infants should be breastfed exclusively during the first six months of life and then breastfed continuously until two years of age or beyond [4]. From six months of age, breast milk should be complemented with a variety of adequate, safe and nutrient-dense foods [4]. For adults, the healthy eating advice from the WHO Regional Office for the Eastern Mediterranean, recently updated to be relevant during the COVID-19 pandemic, includes the recommendation to eat a variety of fresh and unprocessed foods every day—especially fruits, vegetables, pulses, nuts and whole grains—as well as some protein sources (e.g., meat, fish, egg) and healthy fats (e.g., found in fish, avocado, nuts, olive oil, soy, canola, sunflower and corn oils) [5]. Limiting foods containing trans-fatty acids or high in saturated fats, sugars and/or salt, as well as limiting the use of salt in food preparation, is also recommended. This general guidance is also applicable to children over two years old.

Over the last decade, action to improve nutrition in the Region has been guided by the Regional strategy on nutrition 2010–2019 and plan of action [6]. In relation to tackling unhealthy diets efforts were boosted by adoption of a regional framework for action on NCDs, including action to reduce unhealthy diet, in 2012 [7] and a regional framework for action on obesity prevention [8], based on policy priorities previously identified for prevention of obesity and diabetes in the Region [9], in 2018.

This was followed by adoption of a new Strategy on nutrition for the Eastern Mediterranean Region 2020–2030 in October 2019, to support the countries in the Region in strengthening their action on nutrition to achieve food security, end all forms of malnutrition and improve nutrition throughout the life course [1]. In addition to efforts to prevent undernutrition and improve maternal, infant and young child nutrition, the Strategy is intended to support countries in their efforts to ensure universal access to healthy and sustainable diets.

Countries were already working towards globally agreed nutrition targets (including ensuring no rise in overweight among children under five by 2025) [10] and global NCD targets, including to reduce mean population sodium intake by 30% and halt the rise in diabetes and obesity by 2025 [11]. In addition, to measure progress towards these goals, indicators for four specific measures to reduce unhealthy diet—relating to salt reduction, limiting saturated fats and elimination of trans-fatty acids, food marketing to children and marketing of breast-milk substitutes—had been defined [12].

The regional nutrition strategy, adopted in 2019 by the WHO Eastern Mediterranean Regional Committee Meeting, includes a number of specific objectives relating to prevention of overweight/obesity and diet-related NCDs and reducing unhealthy diet:

- Reduce the prevalence of overweight in children under five to not more than 3%
- Halt the rise in diabetes and obesity in adults
- Halt the rise in overweight in school-age children and adolescents 5–18 years old
- Reduce mean population intake of salt/sodium by 30%, and
- Virtually eliminate industrially-produced trans-fatty acids from the food supply.

In addition, the Strategy highlights a number of recommended priority actions relating to reducing unhealthy diets and creating healthy food environments:

- Progressively reduce intakes of salt, sugars and saturated fats by improving the nutritional quality of foods through government-led reformulation programmes
- Implement a tax on sugar-sweetened beverages and use other taxes and subsidies to promote healthy diets
- Review food subsidy programmes and progressively eliminate subsidies for all types of fats/oils and sugars
- Implement mandatory standards for ingredient listing, back-of-pack nutrient declarations and simplified front-of-pack labelling for all pre-packaged foods
- Introduce and enforce mandatory guidelines for provision of healthy food in public institutions, and
- Implement the WHO Set of recommendations on marketing of foods and non-alcoholic beverages to children.

This review aims to (a) present current prevalence and recent trends in overweight and obesity in the Region and (b) summarize current implementation of strategies, policies and interventions to promote consumption of healthy diets and reduce exposure to unhealthy diets across the Eastern Mediterranean Region. This approach is based on and summarizes the progress realized in recent years—through adoption of regulatory, fiscal and voluntary measures—and highlights where further progress in implementation is still needed.

## 2. Materials and Methods

This paper summarizes the prevalence of overweight and obesity and implementation of policies to promote healthy diets in the WHO Eastern Mediterranean Region. The Region includes Afghanistan, Bahrain, Djibouti, Egypt, Islamic Republic of Iran (hereafter Iran), Iraq, Jordan, Kuwait, Lebanon, Libya, Morocco, Pakistan, occupied Palestinian Territory, Oman, Qatar, Kingdom of Saudi Arabia (hereafter Saudi Arabia), Somalia, Sudan, Syrian Arab Republic (hereafter Syria), Tunisia, United Arab Emirates and Yemen.

For this paper, information on the prevalence of overweight, obesity and diabetes in the WHO Eastern Mediterranean Region are summarized. Where available, regional trend data are presented for pre-school children, school-age children and adolescents, and adults. Country trends for overweight and obesity for children and adolescents and for adults are also presented. It should be noted that the periods to present the changes in prevalence over time do vary by age group, reflecting the available data and analyses.

For children under five years old, data on overweight prevalence are derived from the UNICEF/WHO/World Bank Group Joint Child Malnutrition Estimates that have been generated from a country-level dataset which is mainly comprised of estimates from nationally representative household surveys which measured height and bodyweight [13]. Prevalence estimates are based on the 2006 WHO child growth standards, and overweight figures refer to the proportion of children with weight-for-height more than two standard deviations above the median [14]. The methods have been described elsewhere [15] and are collected in line with the operational guidance for the Global Nutrition Monitoring Framework [16].

In relation to children and adolescents between 5 and 19 years of age, estimates were obtained from the NCD Risk Factor Collaboration which are, in turn, based on data provided to WHO and the NCD Risk Factor Collaboration or obtained through a literature review [17]. For those estimates adjustments had been made to standardize risk factor definition, age groups, reporting year and representativeness of the population. Age-standardized prevalence estimates were calculated to adjust for differences in age/sex structure between populations and to enable comparisons between countries. Prevalence of overweight is defined as the percentage of the population with age standardized body mass index (BMI) greater than 1 standard deviation above the median, according to the WHO reference for school-age children and adolescents [17,18,19].

For adults, age-standardized estimates were obtained from the NCD Risk Collaboration, as described above [17]. Overweight is defined as BMI of 25 kg/m^2^ or higher. This includes, therefore, both overweight and obesity (BMI ≥ 30).

Implementation of policies relating to healthy diets in the countries of the WHO Eastern Mediterranean Region is summarized. Data have been extracted from various sources. These include WHO’s global [20] and regional health observatories [21], data collected for the second WHO Global Nutrition Policy Review 2016–2017 [22], WHO reports on implementation of the International Code of Marketing of Breastmilk Substitutes [23] and the Baby-friendly Hospital Initiative [24], the WHO Global database on the Implementation of Nutrition Action (GINA) [25], communication about country-level action from WHO country offices and national government nutrition focal points, and other relevant academic papers or grey literature [9,12,26,27,28,29,30,31,32,33,34,35,36,37,38,39,40,41,42,43,44,45,46,47,48].

Specifically, data was collected on the policy areas related to healthy diet that feature in the new regional nutrition strategy [1]. Policy areas specifically related to maternal, infant and young child nutrition will be reported on separately. The following policy areas were included:

- governance, including multisectoral coordination
- inclusion of promotion of healthy diet and prevention of obesity and diet-related NCDs in recent policies, strategies or plans
- school nutrition and health
- education and information
- nutrition labelling
- elimination of industrially-produced trans-fatty acids from the food supply
- reducing levels of salt, sugars and saturated fats in the food supply
- marketing restrictions
- fiscal measures (taxes and subsidies), and
- food and nutrition surveillance and food composition data.

Data are presented in narrative or tabular form. In relation to policy adoption and implementation data are presented with countries grouped according to country income level. The World Bank classification was used in order to identify income level of each country [49]. The low-income group includes: Afghanistan, Somalia, Sudan, Syria and Yemen. The lower middle-income group includes: Djibouti, Egypt, Morocco, Pakistan, Tunisia and the occupied Palestinian Territory. The upper middle-income group includes: Iran, Iraq, Jordan, Lebanon and Libya. The high-income level includes: Bahrain, Kuwait, Oman, Qatar, Saudi Arabia and United Arab Emirates (UAE).

## 3. Results

The results show that prevalence of overweight and obesity has increased among adults, adolescents and older children across the WHO Eastern Mediterranean Region in recent decades, but that there has not been an increase in the prevalence rate in younger children. Many countries are actively implementing strategies and specific policies or regulatory measures to promote healthy diets and prevent overweight/obesity and diet-related NCDs.

### 3.1. Overweight and Obesity in the Eastern Mediterranean Region

**Figure 1:**
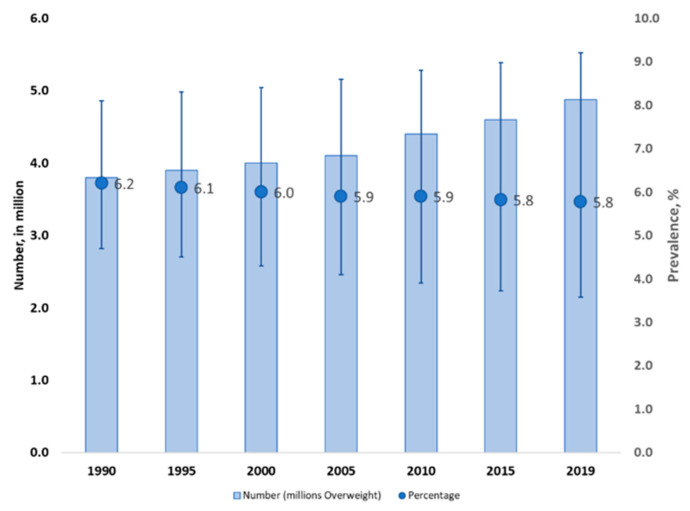
Prevalence of overweight among children under five years of age in the Eastern Mediterranean Region, 1990 to 2019. Source: Data from Unicef, WHO and World Bank 2020 Joint Malnutrition Estimates [13].

Between 1990 and 2019 the prevalence of overweight among children under five years in the Eastern Mediterranean Region declined slightly from 6.2% to 5.8% (Figure 1). Despite this encouraging trend, there is no room for complacency because the numbers of children affected increased from 3.8 million to 4.9 million. Among countries with more recent data (2015 or later), prevalence of overweight in this age group ranges from 2.5% (Pakistan) to 17.2% (Tunisia).

**Figure 2:**
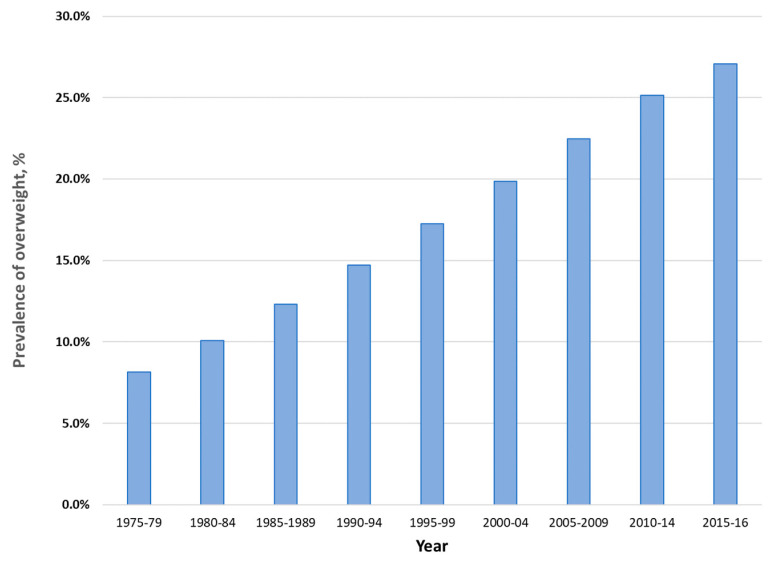
Age-standardized prevalence of overweight and obesity among children and adolescents aged 5 to 19 years in the Eastern Mediterranean Region, 1975 to 2016, both sexes. Data from the NCD Risk Factor Collaboration (http://ncdrisc.org/index.html) [17].

**Figure 3:**
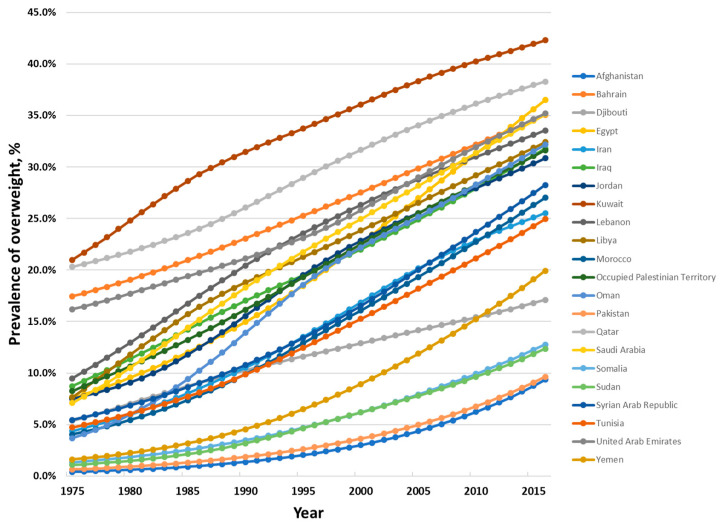
Age-standardized prevalence of overweight and obesity among children and adolescents aged 5 to 19 years in the countries of the Eastern Mediterranean Region, 1975 to 2016, both sexes. Data from the NCD Risk Factor Collaboration (http://ncdrisc.org/index.html) [17].

Among children and adolescents aged between 5 and 19 years, the prevalence of overweight has increased dramatically over the last four and a half decades, from 7.4% in 1975 to 27.4% in 2016 (Figure 2). Country data show few signs of any levelling off in the increase in prevalence in this age group (Figure 3).

**Figure 4:**
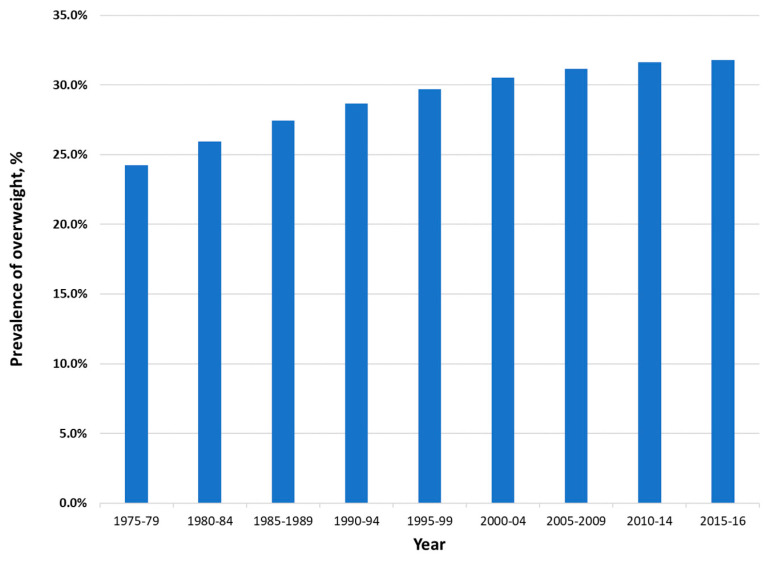
Age-standardized prevalence of overweight and obesity among adults 18 years or older in the Eastern Mediterranean Region, 1975 to 2016, both sexes. Data from the NCD Risk Factor Collaboration (http://ncdrisc.org/index.html) [17].

**Figure 5:**
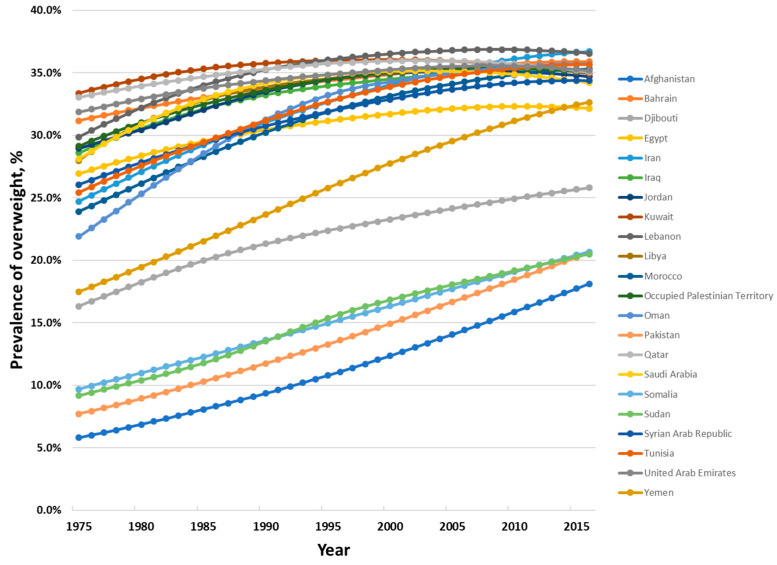
Age-standardized prevalence of overweight and obesity among adults 18 years or older in the countries of the Eastern Mediterranean Region, 1975 to 2016, both sexes. Data from the NCD Risk Factor Collaboration (http://ncdrisc.org/index.html) [17].

Among adults, prevalence of overweight and obesity combined increased from 23.5% in 1975 to 31.8% in 2016 (Figure 4). Trends for individual countries point to the rate of increase levelling off, and even some decreasing prevalence, among some of the countries in the Region with the highest prevalence levels (Figure 5).

### 3.2. Implementation of Policies to Tackle Unhealthy Diet

A wide array of policy options exists to promote healthy diets and tackle unhealthy diet. The options include, for example, the recommended actions identified in the Framework for Action outcome document of the Second International Conference on Nutrition in 2014 [50] and WHO’s package of ‘Best Buys’ and other recommended interventions for reducing unhealthy diet to tackle NCDs [51]. Progress in implementing measures in 10 policy areas is summarized in the following sections.

#### 3.2.1. Nutrition Governance, Multisectoral Coordination and Capacity

Strong government leadership, robust nutrition governance and multisectoral participation are important for effective nutrition action. Strengthening or establishing comprehensive multisectoral coordination mechanisms at various levels, in order to harness all the many different sectors that can play a role in tackling malnutrition, was recommended in the Framework for Action [50].

According to data from the Global Nutrition Policy Review 2016–2017, nine countries (43%) in the Region reported having one coordination mechanism for nutrition and another nine reported having multiple mechanisms [22]. Examples of such coordination mechanisms include national committees, high-level councils of ministers, parliamentary commissions, working groups and task forces. Only three (14%) of countries reported that they did not have such a mechanism. In the majority of cases (88%) the mechanism was located in the Ministry of Health—reflecting the central role of the health sector in improving nutrition, but in 12% of cases it was located in the office of the President or Prime Minister, reflecting the high-level political commitment that is needed to drive action across government.

Countries’ capacity to implement many nutrition-related actions is related to the availability of nutrition professionals—trained nutritionists or dieticians—in the country. In the Eastern Mediterranean Region, 16 countries report having higher education institutions offering training in nutrition [22]. In general, however, there is a low density of nutrition professionals per 100,000 population, with only three countries in the region (Iran, Jordan and Syria) reporting more than 10 nutrition professionals per 100,000. The level varies considerably between countries, ranging from zero to 20.2 (Table 1) [22].

#### 3.2.2. Inclusion of Healthy Diet Promotion and Policy for Prevention of Obesity and Diet-Related NCDs

According to data from the Global Nutrition Policy Review 2016–2017, the vast majority of countries in the Region (89%) had a comprehensive or topic specific nutrition policy [22] and several other countries have published policies or action plans more recently [34,35,41,42,52,53].

In all 22 countries, promotion of healthy diet and/or prevention of obesity and diet-related NCDs have been included in recent policies, strategies or plans [22,35,52,54,55,56].

#### 3.2.3. School Nutrition and Health

Schools have been highlighted as an important setting for opportunities to improve children’s nutrition through ensuring the availability of healthy food and beverages. This includes restricting the provision, sales and promotion of foods that are high in saturated and trans-fatty acids, salt and/or free sugars in school meal programmes, tuck shops in or even around schools, at school events and in the school premises [57,58]. The WHO Nutrition-Friendly Schools Initiative provides a framework for integrated measures to improve health and nutrition through the school setting, which have been widely implemented in the Region. According to the Global Nutrition Policy Review 2016–2017, more than three quarters (79%) of 19 countries in the Region providing information reported having a school health and nutrition programme [22]. These programmes generally aimed to tackle both child undernutrition and overweight/obesity.

Eleven countries had included nutrition in the school curriculum or provided training in nutrition for school staff. Nine countries were providing school meals or implemented school feeding programmes—such programmes can be useful “double-duty” actions which are effective at preventing undernutrition and overweight or obesity. Four countries—Egypt, Jordan, Kuwait and Morocco—reported providing free school meals for all children and Tunisia provides them to disadvantaged children.

Eleven countries in the Region had established rules for foods and beverages available in schools and five countries had banned vending machines from school premises. Countries including Bahrain, Iran, Jordan, Kuwait, Lebanon, Oman, occupied Palestinian Territory, Qatar and Saudi Arabia have banned sales or provision of products such as soft drinks, potato crisps and sweet biscuits in schools [45].

#### 3.2.4. Education and Information

Educating and informing the population about nutrition remain important elements of strategies to improve nutrition. Important government actions include issuing food-based dietary guidelines giving clear advice on healthy diets in the national context and various educational, counselling and awareness-raising activities [50]. Implementation of nutrition education and counselling in different settings to increase the intake of fruits and vegetables and implementation of mass media campaigns on healthy diets are included as WHO recommended interventions to reduce unhealthy diet as part of WHO’s ‘Best Buys’ package [51].

Actions in relation to information and education are widely implemented. By 2017, 11 countries in the Region reported having disseminated dietary guidelines, ten had implemented programmes on nutrition and diet counselling (56%) and seven had implemented media campaigns on healthy diet [22]. A 2019 narrative review identified food-based dietary guidelines from six countries in the Region (Afghanistan, Iran, Saudi Arabia, Lebanon, Oman and Qatar) [36], and United Arab Emirates published guidelines in 2019 [59].

#### 3.2.5. Nutrition Labelling

It is also important to inform and educate the population about the nutritional quality of foods. Implementation of nutrition labelling is recommended as one of WHO’s ‘Best Buys’ [51]. There is increasing momentum for introduction of mandatory simplified front-of-pack labelling [60]. Twelve countries in the Region had introduced rules on nutrition labelling, although only seven have introduced specific rules on nutrition and health claims [22]. Five countries have implemented, or are in the process of implementing, simplified front-of-pack nutrition labelling [26]. Iran, Saudi Arabia and United Arab Emirates have all introduced traffic light labelling—such labels are already mandatory in Iran but have been introduced initially only on a voluntary basis in Saudi Arabia and the United Arab Emirates. By 2017, it was estimated that 80% of food products in Iran carried the traffic light label [61]. Tunisia is introducing a health tick logo for healthier products and Morocco has been testing introduction of the Nutri-Score logo [26].

Some countries have taken steps to improve nutrition information available when consumers eat outside the home. Saudi Arabia, for example, introduced a technical regulation to require calorie content of meals and juices prepared outside the home to be displayed [29] and issued a technical regulation for mandatory inclusion of added sugars on nutrition labels on pre-packaged foods.

#### 3.2.6. Elimination of Industrially-Produced Trans-Fatty Acids from the Food Supply

Elimination of industrial trans-fatty acids through the development of legislation to ban their use in the food chain is recommended as an effective intervention in WHO’s package of interventions to reduce unhealthy diet [51]. By 2017, more than half of the Region’s countries (12) had adopted national policies to limit saturated fatty acids and virtually eliminate industrially produced trans-fatty acids from the food supply [43]. Specifically in relation to trans-fatty acids, by 2019 10 countries had a national policy in place to ban or virtually eliminate industrial trans-fatty acids (Table 2). According to WHO’s 2020 global progress report, Saudi Arabia is the only country in the Region that is considered to have a best practice trans-fatty acid elimination policy in place. WHO defines ‘best practice’ as legislative or regulatory measures that limit industrially produced trans-fatty acids in foods in all settings and are in line with the recommended approach. The two best practice policies for trans-fatty acid elimination are: (i) a mandatory national limit of 2 g of industrially produced trans-fatty acids per 100 g of total fat in all foods; and (ii) a mandatory national ban on the production or use of partially hydrogenated oils as an ingredient in all foods [62]. Saudi Arabia has also introduced a monitoring mechanism for mandatory trans-fatty acid limits [62]. The report also recognizes that Bahrain, Iran and Kuwait have limits on industrially produced trans-fatty acids—although these limits are less restrictive than the recommended approach—and that Jordan, Oman, Pakistan and Tunisia have other complementary measures in place. Afghanistan, Egypt, Lebanon, Morocco, Qatar and United Arab Emirates all have a national policy commitment to eliminate trans-fatty acids [62].

Assessments of sources and intakes have been conducted in Saudi Arabia, Egypt, Lebanon, Jordan, Pakistan, Tunisia, occupied Palestinian Territory, Morocco and Iran [28].

The Iranian government has gradually reduced permitted levels of trans-fatty acids in edible oils over the last 15 years. The standards for corn oil, palm oil, frying oil and mixed liquid oils were revised in 2005 to reduce the maximum level of trans-fatty acids (to less than 10%, down from over 20%). The permitted levels were reduced to less than 2% by 2015 [37,63]. Analyses suggest that the average trans-fatty acid level in frying and liquid oils is now below 1% [47]. For some other products, the upper limit is 5% (e.g., shortening for bakery products) [37]. It is estimated that these measures have resulted in reduction in household use of trans-fatty acids from 168,000 tons to 16.8 tons and in industrial use from 196,000 tons to 28,000 tons [38]. Ministry of Health surveys have also shown that average trans-fatty acid intakes have dropped, but there remains further work to be done to totally eliminate industrially-produced trans-fatty acids from the food supply [64].

For the six countries of the Gulf Cooperation Council (GCC)—namely, Bahrain, Kuwait, Oman, Qatar, Saudi Arabia and the United Arab Emirates—a standard relating to trans-fatty acid elimination was developed and agreed by the Gulf Standards Organization (GSO 2483) in 2015 and became effective in 2016 [65]. The standard specifies a maximum level of 2% in vegetable oils and soft spreadable margarines and 5% in all other foods, including ingredients sold to restaurants, requiring declaration of trans-fatty acids as part of nutrition labels for products containing 0.5 g per 100 g or more and regulating “trans-fat free” claims [65]. The GCC Member States need to approve implementation before the standard can come into effect in the individual countries. In Saudi Arabia, a requirement for trans-fat labelling came into effect in September 2016, and upper limits for trans-fatty acids (as per the GSO standard) came into effect in 2017. This has been followed by a voluntary agreement to limit partially hydrogenated oils (PHO) in food products from December 2018 and a mandatory ban from January 2020 [39,40]. In Oman, a Ministerial Decree has been issued stating that the Directorate of Standards should follow all GSO-approved standards and a Ministerial Decree to prohibit any use of partially hydrogenated oil is in preparation [64]. In Qatar, compliance with GCC technical standards is mandatory and in 2020 the GSO standard 2483 had been submitted to the cabinet for endorsement. In Kuwait, the government issued a resolution adopting the GSO standard in 2017 and a new trans-fatty acid regulation will be implemented from 1 January 2021 [66]. The United Arab Emirates is developing a national version of the GSO standard on trans-fatty acid elimination [64]. The GSO standard was adopted in Bahrain in 2016, and will be enforced on a mandatory basis from 2020, building on previous voluntary initiatives that offered technical support to producers, enabling, for example, bakeries to eliminate trans-fatty acids from their products [64].

In Sudan, legislation has also been developed, through food standards legislation, to limit the trans-fatty acid content of fats and oils, and standards for partially hydrogenated oil and vegetable shortening are being updated [64]. In Jordan, a multi-sectoral committee is addressing trans-fatty acid elimination and, with WHO support, has prohibited the use of plant oils and fat in dairy products, banned trans-fatty acids in public sector hospital food and developed a legislative proposal for industrial trans-fatty acid elimination for consideration by the food standards and specification authority.

In Egypt, the Ministry of Health has drafted a roadmap for action on industrial trans-fatty acid elimination, with participation of all sectors [64]. Some factories are reported to have started using esterification technology, on a voluntary basis, to eliminate the hydrogenation process.

In Pakistan, a technical working group has been established [62] and awareness activities have been organized to increase understanding across multiple sectors, including among parliamentarians, of the need to take action on trans-fatty acids [64]. Responsibility for formulating and enacting trans-fatty acid legislation has recently been transferred from provincial authorities to the Pakistan Standards and Quality Control Authority and a national action plan for industrial trans-fatty acid elimination is being developed [64]. Currently there are some limits on trans-fatty acids in particular food products at provincial level, and these vary between provinces [64].

A number of other countries in the Region—including Morocco, occupied Palestinian Territory and Syria—are developing regulatory measures to eliminate industrial trans-fatty acids and are at different stages of the legislative process [64]. Other countries in the Region—such as Afghanistan and Lebanon—are preparing broad nutrition and/or food policy documents, which will include the issue of trans-fatty acids [64].

#### 3.2.7. Reducing Levels of Salt, Sugars and Saturated Fats in the Food Supply

Reducing salt intake through the reformulation of food products is recommended as a WHO Best Buy for tackling NCDs by reducing unhealthy diet [51]. Thirteen countries had fully or partially adopted national policies to reduce population salt/sodium consumption by 2019 (Table 2) [43].

A number of countries have taken specific action to reduce salt levels in bread, which is a major contributor to salt intakes across the Region. In 2019, Saudi Arabia set a mandatory upper limit for salt in bread (1%) [29]. Iran has introduced legislation to limit salt levels in bread, albeit to a level (1.8%) higher than that recommended by the WHO Regional Office (0.5% or 1% depending on the context) [27]. Oman and the United Arab Emirates have reviewed food standards and set a mandatory maximum level for salt in bread and established a benchmark goal of 0.5% [27]. Kuwait and Qatar have achieved a reduction of at least 20% in the salt levels in bread since 2013/4 by working with the main supplier [27]. Jordan and Bahrain have prepared legislation and set benchmarks. In the occupied Palestinian Territory, cooperation with bakeries was initiated in 2016 and an upper limit on salt content in bread (0.9%) has been established. In Morocco, where the level of salt in bakery-produced bread was found to be very high (17.8 g/kg flour), the government established a programme to reduce salt in bakery bread and obtained commitments from the National Federation of Bakers to reduce the salt levels by 2020. A 2016 evaluation in Casablanca found that the average amount of salt in bakery bread was 13.1 g/kg compared to 17.8 g/kg in 2014 [33]. Similar results were found in Fez (12.3 g/kg). In 2018, a decree fixing an upper limit for salt in bread was submitted for approval.

Iran’s national salt reduction strategy covers other food groups and the permitted level of salt has been reduced for snacks (from 2.5% to 1.5%), canned tomato paste (3% to 2%), and potato chips (1.5% to 1%), as well as bread (2.3% to 1.8%). Further revisions to standards for other products are planned [63]. In Kuwait, a Salt and Fat Intake Reduction Task Force was established in 2013 and has drawn up voluntary agreements to gradually reduce the salt content of cheese, as well as bread [63]. In Saudi Arabia, guidelines for voluntary reformulation to reduce salt levels have been introduced and a voluntary reformulation initiative—with international and local food establishments and importers, and targeting sugars, salt, saturated fat and trans-fatty acid reductions—has been established [29].

#### 3.2.8. Marketing Restrictions

Advertising and marketing increase intakes and preferences for energy-dense and nutrient-poor foods and beverages [67], and such marketing is often targeted at children. WHO recommends restricting the marketing of foods and non-alcoholic beverages to children to reduce the impact on children of advertising and other forms of promotion for food and drink products high in saturated fatty acids, trans-fatty acids, sugars or salt/sodium [68].

In 2019, four countries in the region reported the existence of any policy on marketing of foods to children (Table 2). No country in the Region has adopted comprehensive policies restricting marketing of unhealthy food to children [45]. Nonetheless, there is growing awareness of the need to take action on this issue and there has been some progress in the Region [45].

Iran, for example, has taken substantial steps to restrict the marketing of foods designated as unhealthy [45]. Broadcast advertising (television and radio) of soft drinks has been prohibited since 2004. The regulations have been designed to cover both children (under 12 years) and adolescents (between 12 and 19 years) on the grounds that both these groups are susceptible to the adverse effects of unhealthy food marketing. The sale of unhealthy food in school canteens and by vendors around schools is also prohibited. There are also restrictions on the sponsorship of some social events, such as seminars, congresses and food festivals, by the food industry that produces unhealthy food (e.g., soft drinks, edible oils and salty snacks). In 2014, the Ministry of Health and Medical Education proposed a list of 24 food items to be prohibited from advertising in all media [45].

Some other countries have started to align their national regulations with the WHO Recommendations or have considered how they could do so [45]. Egypt has issued a Ministerial Decree to prohibit the marketing of unhealthy food on governmental television and radio. Saudi Arabia has issued a law to prohibit the marketing of energy drinks and the Ministry of Health has asked the official media not to promote unhealthy food to children. Oman has carried out a study on the extent of marketing of unhealthy foods to children and is developing legislation to restrict unhealthy food marketing to children [45]. Tunisia has also drafted a legislative proposal to restrict unhealthy food marketing to children on television or the internet and at games, sports events and cultural events. A number of countries—including Bahrain, Iran, Kuwait, Lebanon, Morocco, Oman and Tunisia—have adopted the Regional Nutrient Profile Model [69] to identify foods for which marketing to children should be restricted [45].

#### 3.2.9. Fiscal Measures—Taxes and Subsidies

Taxes and subsidies which affect the relative price of healthy and unhealthy products have a role to play in promoting healthy diets. The WHO Regional Office for the Eastern Mediterranean recommends implementation of a tax on sugar-sweetened beverages [8] and effective taxation on sugar-sweetened beverages is included as an effective intervention in the package of measures to tackle NCDs by reducing unhealthy diet [51]. By the end of 2019, taxes on different categories of non-alcoholic drinks had been introduced in Bahrain, Iran, Saudi Arabia, Morocco, Oman, Qatar, Tunisia and United Arab Emirates (Table 2). A 50% tax on carbonated drinks and 100% tax on energy drinks was adopted for Gulf Cooperation Council (GCC) countries in 2016 and Saudi Arabia was the first to implement the measure in June 2017. In addition, a 5% value added tax was added to the beverage tax in 2018 and, since December 2019, a 50% tax has also been applied to other sugar-sweetened beverages. Soda prices increased by 67% and annual purchases, in volume per capita, of soda and energy drinks reduced by 41% and 58% respectively in 2018 compared to 2016 [31].

The Eastern Mediterranean Region has had a long tradition of subsidizing food prices for consumers. Such subsidies are not always aligned with healthy diets, with some countries subsidizing sugar, oils and white bread. Some countries in the Region have now eliminated subsidies for sugar and/or fats and oils [9].

#### 3.2.10. Food and Nutrition Surveillance and Food Composition Data

Establishment of food and nutrition surveillance is important to generate data to direct policy action and interventions, and to monitor progress towards agreed goals. A food and nutrition surveillance system should provide regular and timely collection, analysis and reporting of data on nutrition risk factors, nutritional status and nutrition-related diseases in the population.

By 2017, eight countries in the Region—Afghanistan, Kuwait, Oman, occupied Palestinian Territory, Qatar, Sudan, Syria and Yemen—had developed nutrition surveillance systems [43] and a number of countries in the Region—including Bahrain, Iraq, Kuwait and Tunisia—have conducted large scale nutrition surveys in the last decade, or have adopted a sentinel site approach.

In order to monitor population nutrition intakes and to issue healthy eating guidelines, it is also important to have up-to-date information on the nutrient composition of foods. In relation to promoting healthy diets, this is particularly important for identifying key dietary sources of saturated and trans-fatty acids, free sugars and salt. In 2017, seven countries in the Region—Egypt, Kuwait, Lebanon, Libya, Oman, Pakistan, Tunisia—reported having a national food composition table or database [22]. This is an area where substantial progress has been made during the last decade. A partnership between the WHO Regional Office and the Quadram Institute in the UK, funded by the UK Medical Research Council, provided extended technical and scientific support to countries to develop a national food composition table or database. Researchers from 11 countries participated in the project and benefited from training and capacity development in the use of improved standardized methodologies to update food composition data. Nine countries in the Region—Egypt, Iran, Iraq, Jordan, Kuwait, Morocco, Pakistan, Sudan and Tunisia—have now progressed with their national food composition database and developed knowledge and capacity to further progress development of these tables [32].

## 4. Discussion

A major strength of the part of this study presenting overweight and obesity data (Section 3.1) is that the data are drawn from well-established, verified, quality global sources [13,17]. Furthermore, bodyweight and height data were measured, thus avoiding the biases associated with self-reported data. Nonetheless, the methods used to generate these data are subject to the same strengths and weaknesses as other malnutrition data. Prevalence estimates have been generated mainly on the basis of estimates from nationally representative household surveys and there are gaps in available data. A strength of the review in relation to data on implementation of policies (Section 3.2) is that the data were largely drawn from well-established, verified sources [20,21,22,23,24] and supplemented with additional information from the academic and grey literature. One weakness of this approach is that it relies on reports on the adoption or existence of policies, strategies or mechanisms, but does not attempt to assess how well coordination mechanisms are working, how effectively policies or mechanisms have been implemented or their impact on the ground. In general, there is a need for much better evaluation data to assess the impact of policy measures and other interventions, as well as the operational challenges in their implementation and any unintended consequences.

The findings presented in Section 3.1 show that prevalence of overweight and obesity has increased among adults, adolescents and older children across the WHO Eastern Mediterranean Region in recent decades. The number of children under five who are affected by overweight has also increased, although the proportion of children affected has declined slightly. Among the countries with the highest prevalence rates, there are signs that the increase is slowing down or even that prevalence is declining, but the rates remain high. These data clearly point to an urgent need for all countries in the Region to address the issue of obesity prevention and to implement strategies, policies and other measures to promote healthy diets and prevent unhealthy weight gain.

Widespread adoption of nutrition policies and strategies that include actions to promote healthy diets and prevent obesity and diet-related NCDs, along with the frequent existence of multisectoral coordination mechanisms, indicates strengthened political commitment to improve nutrition in the countries of the Eastern Mediterranean Region. There has also been progress in areas which are important to enable action on healthy diets, such as the establishment of up-to-date, comprehensive food composition data.

In order to meet existing global and regional goals and targets [6,10,11,70,71], however, there is a need to scale up such actions across the Region. There is evidence of considerable momentum behind efforts to eliminate industrial trans-fatty acids from the food supply across the Region, but this will have to be maintained and accelerated to be able to meet WHO’s goal of virtually eliminating industrial trans-fatty acids by 2023 [71]. Similarly, implementation of fiscal measures, particularly taxes on sugar-sweetened beverages has accelerated, but still only applies to a minority of the Region’s population. Although actions to educate and inform the public about nutrition are widely implemented, there remains a need to expand implementation of rules on nutrition labelling and to scale up introduction of simplified front-of-pack nutrition labelling. The scope of government-led reformulation programmes must be expanded considerably beyond the current focus on salt and, in particular, bread in order to achieve meaningful reductions in intakes of salt, saturated fats and free sugars.

Furthermore, there are areas where progress in the Region is lacking. Concrete policy action to restrict children’s exposure to marketing for foods and non-alcoholic beverages high in fats, sugars or salt is rare. While around half of the Region’s countries are implementing measures to improve the nutritional quality of food in schools, the considerable potential to increase access to healthy diets by setting nutrition standards for food served in other public institutions—such as hospitals, child care and older people care facilities, government offices and military establishments—currently remains untapped.

The countries in the Region face a number of barriers and challenges which can hinder their efforts to promote healthy diets and prevent obesity and diet-related NCDs. The Eastern Mediterranean Region is characterized by diverse socioeconomic status and health challenges. The income level of countries ranges from among the world’s highest to lowest, and there are also huge wealth and health inequalities within countries. In 2019, nearly two-thirds of countries in the Region were dealing with socio-political or economic crises or armed conflict. Food insecurity has greatly increased in conflict-affected countries, resulting in the world’s largest food crisis in Yemen.

Specifically in relation to nutrition, to date there has been underinvestment in nutrition and there is a general shortage of human capacity, with a low density of trained nutrition professionals in most countries [1]. There is clearly recognition of the importance of multisectoral action on nutrition, but, in practice, the role of sectors other than health generally remains underdeveloped. Many countries in the Region are heavily dependent on imported foods and food marketing is often disseminated across borders via pan-regional media, creating particular challenges. In addition, policymakers worldwide face opposition from parts of the food industry that seek to hinder their efforts to regulate or introduce taxes [72], which can undermine political will to implement robust measures. Furthermore, where regulatory measures are adopted and enacted, their impact can be diminished by weak or under-resourced enforcement mechanisms.

In addition to these barriers, and the ongoing crises throughout the Region, the COVID-19 pandemic threatens to further undermine food security and nutrition in various ways [73]. People with COVID-19 who are affected by obesity or have underlying diet-related NCDs, such as cardiovascular disease, diabetes or cancer, have an elevated risk of becoming severely ill or dying [74,75,76,77]. The combined effects of COVID-19 and the measures taken to mitigate its impact, along with the emerging global recession, threaten to disrupt food systems and potentially reduce accessibility and affordability of safe and nutritious foods [78,79]. In addition to their impact on food insecurity and undernutrition, social and economic consequences of COVID-19 may have a negative impact on healthy diets. Healthy diets cost five times more than a diet that simply meets energy needs through a starchy staple and 60% more than the cost of a diet that meets minimum nutrient requirements [79]. Nutritious foods such as fruits, vegetables and animal source foods typically cost more than starchy staples, oils, sugars and energy-dense foods high in fats, sugars and/or salt [79]. In times of economic hardship, therefore, families may shift away from nutritious foods to cheaper, energy-dense foods of minimal nutritional value [79]. In addition, due to movement restrictions, people are having to spend more time at home and there may be a switch to more sedentary lives and less healthy diets—with more fast foods or highly-processed foods high in fats, sugars and salt and fewer fresh foods, including fruits and vegetables. The WHO Regional Office for the Eastern Mediterranean issued clear nutrition advice for adults during the COVID-19 outbreak [5] and, more generally, healthy eating advice from WHO highlights healthy and affordable foods that are available in the Region [80]. Dishes based on legumes (beans, lentils and chickpeas), such as bean stews, are particularly popular in the region, and these can be good affordable sources of protein, fiber and micronutrients [80]. In the long-term, it is also conceivable that the pandemic could exert effects on overweight and obesity among the population through disruptions to the food supply or to delivery of health interventions that undermine optimal infant and young child feeding. Breastfeeding reduces the child’s risk of overweight/obesity and type 2 diabetes later in life [81] and is protective against some cancers and type 2 diabetes in mothers [82]. WHO has updated advice on breastfeeding and infant feeding in the context of COVID-19 for the Eastern Mediterranean Region [83].

These interactions between nutrition early in life and later health outcomes, and between different forms of malnutrition, underline the importance of action to prevent all forms of malnutrition. A comprehensive, multisectoral approach underpins the United Nations Decade of Action on Nutrition 2016–2025 and the WHO Strategy on nutrition for the Eastern Mediterranean Region 2020–2030, and this can be readily adapted to the new context of the COVID-19 crisis.

## 5. Conclusions

Prevalence of overweight and obesity has increased among adults, adolescents and older children across the WHO Eastern Mediterranean Region in recent decades. Among the countries with the highest prevalence rates, there are signs that the increase is slowing down or even that prevalence is declining. There has not been an increase in the prevalence rate in younger children, although the absolute number of children under five years affected by overweight has increased.

The last decade has seen a step up in action to tackle unhealthy diets across the WHO Eastern Mediterranean Region, as countries are increasingly faced with the double burden of malnutrition. This has included efforts to inform and educate the population about healthy diets, by, for example, disseminating guidelines on healthy eating. Crucially, it has also included measures to create healthier food environments, notably by improving the nutritional quality of foods served or available in schools, implementing legislation to virtually eliminate industrial trans-fatty acids, driving salt reduction strategies and applying taxes to sugar-sweetened beverages. There has also been success in updating food composition data, including data on trans-fatty acids, sugars and salt, in a number of countries.

In order to meet the agreed global and regional goals relating to nutrition and diet-related NCDs, countries will need to build on this progress and scale up action across the region while intensifying efforts in areas where concrete action is lacking.

## Figures and Tables

**Table 1:** Density of nutrition professionals in countries of the Eastern Mediterranean Region. (Trained nutritionists or dieticians per 100,000 population) ^1^.

Country	Trained Nutritionists or Dieticians per 100,000 Population
Low-income countries	
Afghanistan	0.1
Somalia	n.d.
Sudan	2
Syria	10.7
Yemen	0.4
Lower-middle income countries	
Djibouti	0.8
Egypt	n.d.
Morocco	1.1
Pakistan	n.d.
occupied Palestinian Territory	n.d.
Tunisia	8
Upper-middle income countries	
Iran	20.2
Iraq	0
Jordan	19.9
Lebanon	n.d.
Libya	3.5
High-income countries	
Bahrain	1
Kuwait	5.8
Oman	5
Qatar	6
Saudi Arabia	6.6
United Arab Emirates	7.6

n.d. = no data; ^1^ Source: Data from WHO Global Nutrition Policy Review 2016–2017 [22].

**Table 2:** Existence of interventions to promote healthy diet in the countries of the WHO Eastern Mediterranean Region.

Countries	Any Policies on Marketing of Food to Children ^1^	Any Policies to Reduce Salt Consumption ^1^	National Policies on Saturated Fatty Acids/Trans-Fatty Acids ^1^	National Policies on Saturated Fats ^1^	National Policies on Trans-Fatty Acid Elimina-tion ^1^	Specific Measure to Ban or Virtually Eliminate Industrial Trans-Fatty Acids ^2^	Tax on Sugar-Sweetened Beverages (Level of Tax Levied) ^3^
Low-income countries							
Afghanistan	x	x	✔	x	x	x	x
Somalia	n.d.	n.d.	x	n.d.	x	x	x
Sudan	x	x	x	x	x	x ^5^	x
Syria	x	x	x	x	x	x	n.d.
Yemen	x	x	x	x	x	x	n.d.
Lower-middle income countries							
Djibouti	n.d.	x	x	x	x	x	x
Egypt	x	✔	x	x	x	x ^4,5^	n.d.
Morocco	✔	✔	✔	✔	✔	x ^4^	✔ (50%)
Pakistan	x	x	x	x	x	x ^8^	x
Occupied Palestinian Territory ^6^	x	✔	✔	n.d.	x	x ^4,5^	x
Tunisia	x	✔	✔	✔	✔	x ^4,8^	✔
Upper-middle income countries							
Iran	✔	✔	✔	✔	✔	✔	✔ (20%)
Iraq	x	✔	✔	✔	n.d.	x	x
Jordan	x	✔	✔	x	✔	x ^8^	x
Lebanon	x	x	x	x	x	x	x
Libya	x	x	x	x	x	x	x
High-income countries							
Bahrain	✔	✔	✔	✔	✔	✔^7^	✔ (50%)
Kuwait	x	✔	✔	✔	✔	✔^7^	x
Oman	✔	✔	✔	✔	✔	x ^5,7,8^	✔ (50%)
Qatar	x	✔	✔	✔	✔	x ^5,7^	✔ (50%)
Saudi Arabia	x	✔	✔	✔	✔	✔ ^7^	✔ (50%)
UAE	x	✔	✔	✔	✔	x ^5,7^	✔ (50%)

^1^ Global Health Observatory noncommunicable disease data indicators (https://apps.who.int/gho/data/view.main.2473); ^2^ Countdown to 2023: WHO report on trans-fat elimination 2020 and information from nutrition focal points ^3^ Information from country nutrition focal points on existence of a tax and, where available, the level of tax levied, e.g., 50% of pre-tax price; ^4^ Assessment has been conducted to identify sources and intakes; ^5^ National action plan or legislation under development; ^6^ All Palestinian data obtained from Ministry of Health; ^7^ The Gulf Standards Organization adopted GSO standard 2483/2015 for the members of the Gulf Cooperation Council—implementation varies between countries (see text); ^8^ Other complementary measures in place; n.d. = no data. ✔ = policy/measure reported, x = no policy/measure reported.
